# Association of Demographic and Socioeconomic Indicators With the Use of Wearable Devices Among Children

**DOI:** 10.1001/jamanetworkopen.2023.5681

**Published:** 2023-03-30

**Authors:** Ethan H. Kim, Jessica L. Jenness, Adam Bryant Miller, Ramzi Halabi, Massimiliano de Zambotti, Kara S. Bagot, Fiona C. Baker, Abhishek Pratap

**Affiliations:** 1 Krembil Centre for Neuroinformatics, Centre for Addiction and Mental Health, Toronto, Ontario, Canada; 2 Department of Psychiatry and Behavioral Sciences, University of Washington, Seattle; 3 RTI International, Research Triangle Park, North Carolina; 4 University of North Carolina at Chapel Hill; 5 Center for Health Sciences, SRI International, Menlo Park, California; 6 Addiction Institute, Department of Psychiatry, Icahn School of Medicine at Mount Sinai, New York, New York; 7 Department of Psychiatry, University of Toronto, Toronto, Ontario, Canada; 8 Vector Institute for Artificial Intelligence, Toronto, Ontario, Canada; 9 King’s College London, London, United Kingdom; 10 Department of Biomedical Informatics and Medical Education, University of Washington, Seattle

## Abstract

**Importance:**

The use of consumer-grade wearable devices for collecting data for biomedical research may be associated with social determinants of health (SDoHs) linked to people’s understanding of and willingness to join and remain engaged in remote health studies.

**Objective:**

To examine whether demographic and socioeconomic indicators are associated with willingness to join a wearable device study and adherence to wearable data collection in children.

**Design, Setting, and Participants:**

This cohort study used wearable device usage data collected from 10 414 participants (aged 11-13 years) at the year-2 follow-up (2018-2020) of the ongoing Adolescent Brain and Cognitive Development (ABCD) Study, performed at 21 sites across the United States. Data were analyzed from November 2021 to July 2022.

**Main Outcomes and Measures:**

The 2 primary outcomes were (1) participant retention in the wearable device substudy and (2) total device wear time during the 21-day observation period. Associations between the primary end points and sociodemographic and economic indicators were examined.

**Results:**

The mean (SD) age of the 10 414 participants was 12.00 (0.72) years, with 5444 (52.3%) male participants. Overall, 1424 participants (13.7%) were Black; 2048 (19.7%), Hispanic; and 5615 (53.9%) White. Substantial differences were observed between the cohort that participated and shared wearable device data (wearable device cohort [WDC]; 7424 participants [71.3%]) compared with those who did not participate or share data (no wearable device cohort [NWDC]; 2900 participants [28.7%]). Black children were significantly underrepresented (−59%) in the WDC (847 [11.4%]) compared with the NWDC (577 [19.3%]; *P* < .001). In contrast, White children were overrepresented (+132%) in the WDC (4301 [57.9%]) vs the NWDC (1314 [43.9%]; *P* < .001). Children from low-income households (<$24 999) were significantly underrepresented in WDC (638 [8.6%]) compared with NWDC (492 [16.5%]; *P* < .001). Overall, Black children were retained for a substantially shorter duration (16 days; 95% CI, 14-17 days) compared with White children (21 days; 95% CI, 21-21 days; *P* < .001) in the wearable device substudy. In addition, total device wear time during the observation was notably different between Black vs White children (β = −43.00 hours; 95% CI, −55.11 to −30.88 hours; *P* < .001).

**Conclusions and Relevance:**

In this cohort study, large-scale wearable device data collected from children showed considerable differences between White and Black children in terms of enrollment and daily wear time. While wearable devices provide an opportunity for real-time, high-frequency contextual monitoring of individuals’ health, future studies should account for and address considerable representational bias in wearable data collection associated with demographic and SDoH factors.

## Key Points

**Question:**

Does the large-scale usage of wearable devices in children vary based on demographic and socioeconomic indicators?

**Findings:**

In this cohort study of 10 414 children, there was a statistically significant association between participants’ sociodemographic characteristics and willingness to enroll and engage in a wearable device study. Black children and those from lower socioeconomic status households were less likely to participate and wore devices for significantly less time than White children and those from higher socioeconomic status households, respectively.

**Meaning:**

The findings of this study suggest that without factoring in the broader social determinants of health that may affect individual and group experiences and participation in research, inequities in data collection using wearable technologies may continue to exist, especially for youths belonging to racial and ethnic minority groups.

## Teaser

This cohort study examines whether demographic and socioeconomic indicators are associated with willingness to join a wearable device study and adherence to wearable data collection in children.

## Introduction

Over the last decade, consumer-grade wearable devices have gained popularity, with as many as 1 in 5 adults in the United States wearing smartwatches or fitness trackers.^1^ With lower costs and increasingly high-fidelity multimodal sensing, wearable devices can help assess the impact of frequent changes in physiological and behavioral patterns on personalized health outcomes.^2^

Researchers have used wearable devices across various health conditions and contexts, including monitoring patients with advanced cancer,^3^ daily stress,^4^ depression,^5^ and autism^6^; patient care management^7,8^; and aiding early detection of COVID-19.^9^ Given the high-frequency daily usage of mobile technologies, with smartphone ownership increasing from 69% to 91% between the ages of 11 and 18 years,^10^ wearable devices are particularly well-matched to better understanding youth health outcomes. Specifically, wearable devices could help to provide in-depth data on sleep and physical activity—important transdiagnostic risk factors for pediatric mental health disorders.^11,12,13^

However, despite the promise of wearable devices in health research, questions remain regarding potential biases in device deployment, data collection, and interpretation.^14,15,16,17^ There is limited evidence on whether long-term health monitoring using wearable devices will yield reliable and equitable real-world data^18^ across the diverse socioeconomic and demographic^19,20^ population spectrum,^21,22^ particularly among racial and ethnic minority groups.^23^ Research shows that social determinants of health (SDoHs)^24^ could potentially affect the acceptability of technology usage among racial and ethnic minority populations. The use of wearable devices in health research should also be evaluated in the context in which devices are worn daily. Fewer in-school resources,^25^ less safe housing and venues for physical activity,^26^ and lower socioeconomic status (SES) may lead to racial and ethnic minority youths spending more time on screen or media activities^10^ compared with their White counterparts. Racial and ethnic minority parents are also more likely to monitor their children’s device usage,^10^ so parental background and views on devices may affect the youth’s willingness or ability to engage.

Prior research also shows multiple structural and systemic barriers^27^ that may significantly affect the self-initiation of personal use of wearable devices among racial and ethnic minority groups. These include, but are not limited to, inequitable allocation of resources and access to health care technology,^26,28,29^ concerns about data accuracy^30^ and privacy,^25^ and language barriers.^31^ In addition, nonuniformity in willingness to join wearable device studies and share health data continually with frequent charging requirements and variation in sensor accuracy may also influence the equitable and representative collection of wearable device data from a large population.^32,33,34,35^ With research using consumer-grade wearable devices still in the early stages, there is a critical need to examine whether data from such devices can be collected equitably across large populations with varying age groups, SES, SDoH factors, and racial and ethnic backgrounds.

To empirically assess some of the challenges associated with large-scale wearable device data collection in children, we used the data collected from devices in the ongoing Adolescent Brain and Cognitive Development (ABCD) Study. The ABCD Study is the most extensive study of brain development and child health in the United States, with more than 11 000 children aged 9 to 10 years old recruited from 21 sites across the United States.^36^ In addition to episodic assessments consisting of neuroimaging, clinical interviews, and neuropsychological tests, the study used wearable devices during the year 2 (Y2) time point, collecting as much as 3 weeks of physiological data. To further understand factors potentially affecting wearable device data collection for children in naturalistic settings, we investigated the following specific questions: (1) can demographic and socioeconomic factors affect large-scale deployment and collection of wearable device data from children? and (2) is the difference in device wear time associated with children’s and their parents’ demographic and socioeconomic characteristics?

## Methods

### Study Design

We used the 4.0 data release from the ABCD Study^36^; specifically, data from 10 414 children enrolled at Y2 follow-up were included in the present analysis. As part of the main ABCD Study, parents provided written consent and children provided assent to participate, with centralized institutional review board approval obtained from the University of California San Diego’s institutional review board.^37^ The study recruited children from schools throughout the United States based on stratified probability^38^ to match the demographic and socioeconomic diversity of the United States.^39^ Further details on the ABCD Study protocol can be found in Karcher et al.^38^ This secondary cohort study followed the Strengthening the Reporting of Observational Studies in Epidemiology (STROBE) reporting guidelines and was approved by the research ethics board of the Center of Addiction and Mental Health (Toronto, Canada).

### Primary Measures

#### Sociodemographic Characteristics

During onboarding and Y2 follow-up, children and their parents completed questions related to demographic and socioeconomic indicators (eg, race and ethnicity, age, gender, height, weight, income, education, marital and employment status). We also used the national percentile value of the area deprivation index (ADI)^40^ to account for variation in participants’ SDoH. ADI is a composite metric that helps compare US neighborhoods based on multiple socioeconomic indicators, including income, education, employment, and housing quality. ADI has been used to compare variations in health delivery and services, especially for the most disadvantaged neighborhood groups.^41^ The study recruitment sites were divided into 5 key geographical regions in the United States, depending on their location. eAppendix 1.1 in Supplement 1 includes more details on demographic and socioeconomic variables.

#### Wearable Device Data

As part of the Y2 follow-up, all participants were given the option to wear a device (Charge HR 2 [Fitbit]). Children who gave their assent and whose caregivers consented were given a device and instructed to wear it continuously, excluding bathing or water activities, over 3 weeks. Automated alerts were sent to participants if 3 days had passed without a sync or if 2 days had passed and the device was not sufficiently charged. If data-sharing issues persisted, research assistants reached out to participants. eAppendix 1.2 in Supplement 1 provides further details on device deployment and data collection.

### Statistical Analysis

#### Demographic Analysis

Statistically significant differences in participants’ sociodemographic characteristics in different subcohorts were assessed using univariate χ^2^ and Kruskal-Wallis tests for categorical and continuous variables, respectively. Rank-based (Spearman) method was used to evaluate correlations between variables of interest.

#### Retention Analysis

Participant retention in the wearable device substudy was assessed based on the last date participants’ device data were shared within the 21-day study observation period. Kaplan-Meier (KM) survival curves^42^ to assess retention differences across sociodemographic and economic factors. The statistically significant differences in retention KM curves were evaluated using nonparametric log-rank test.^43^ A stratified version of the log-rank test was used to adjust for potential heterogeneity across study sites. With participant retention assessed in a short observation period (21 days) based on passively collected wearable data, we used the time to retain 75% of the cohort for comparing retention differences in variables of interest. eAppendix 1.3 in Supplement 1 includes further details on retention analysis.

#### Wear-Time Analysis

Per-minute heart rate (HR) data obtained from devices were used to approximate the total daily wear time (in hours) by calculating the time points for which the device recorded per-minute HR. Previous studies have used HR as a proxy for wear time^22^ such that time intervals with no acquired HR data were regarded as nonwear periods.^44^ We determined the first date of available data as the start of the 3-week observation period per participant. For all participants, day 1 was excluded due to varying start times of device wear, resulting in as many as 20 days of data being used for the present wear-time analysis. Given the multilevel structure of the data, we used a mixed-effects regression model^45^ to investigate potential associations between total wear time and participants’ demographic and socioeconomic factors. The key variables of interest, such as participants’ race and ethnicity, gender, parent’s marital status, education level, ADI percentile, and household income, were considered primary covariates (referred to as fixed effects) in the mixed-effects model. We used random intercepts for recruitment sites and study enrollment quarters to account for the potential clustering of participants within the same recruitment site or enrollment patterns during the COVID-19 pandemic. Mixed-effect models were implemented using lme4 package version 1.1-28.^46^ Statistical significance of the association of variables of interest with wear time was assessed using Satterthwaite approximation method, available from lmerTest package version 3.1-3.^45^ The best model fit was determined based on the Akaike information criterion. Variation inflation factor was used to assess model collinearity for all model covariates and met acceptable thresholds (<5).^47^ For sensitivity analysis, we compared the inference drawn from the retention and wear-time data collected from participants recruited before and during the COVID-19 pandemic. All analyses were performed using either R version 4.0.5 (R Project for Statistical Computing)^48^ or Python version 3.9.7 (Python Software Foundation). Statistical significance was assumed when with a 2-sided *P* < .05.

## Results

### Cohort Characteristics

The mean age (SD) of the 10 414 children enrolled when wearable devices were offered to all active participants was 12.00 (0.72) years. Of these, 5444 (52.3%) were male, and 5615 (53.9%) were White, with the largest racial and ethnic minority groups identifying as Hispanic or Latino (2048 [19.7%]), followed by Black (1424 [13.7%]). A notable proportion of parents (2349 [22.6%]) reported their annual household income to be less than $49 999, with nearly a majority (4599 [44.2%]) earning more than $100 000. Table 1 and eAppendix 2.1 in Supplement 1 further summarize the demographic characteristics of children and parents, respectively. No significant differences in sociodemographic characteristics of the participants were observed between those enrolled in the wearable device substudy during the COVID-19 pandemic (694 of 7424 participants [9.4%]) compared with participants enrolling in the study before COVID-19 (6546 participants [88.2%]) (eAppendix 2.2 in Supplement 1).

**Table 1.** Adolescent Brain and Cognitive Development Study Year 2 Cohort Demographic Characteristics

Characteristic	Participants, No. (%)			*P *value	Test performed (test value)
	Overall	Wearable data availability			
		No device	Device		
No.	10 414	2990	7424	NA	NA
Age, mean (SD), y	12.00 (0.72)	12.11 (0.72)	11.96 (0.72)	<.001	Kruskal-Wallis (88.453; *df* = 1)
Race and ethnicity					
American Indian, Alaska Native, or Pacific Islander	208 (2.0)	50 (1.7)	158 (2.1)	<.001	χ^2^ (208.37; *df* = 6)
Asian	201 (1.9)	64 (2.1)	137 (1.8)		
Black	1424 (13.7)	577 (19.3)	847 (11.4)		
Hispanic	2048 (19.7)	694 (23.2)	1354 (18.2)		
Multiple racial and/or ethnic groups	807 (7.7)	255 (8.5)	552 (7.4)		
White	5615 (53.9)	1314 (43.9)	4301 (57.9)		
Other^a^	111 (1.1)	36 (1.2)	75 (1.0)		
Gender					
Male	5444 (52.3)	1605 (53.7)	3839 (51.7)	.08	χ^2^ (6.7031; *df* = 3)
Female	4955 (47.6)	1384 (46.3)	3571 (48.1)		
Other^b^	9 (0.1)	0 (0.0)	9 (0.1)		
Do not know or refused to answer	4 (0.0)	1 (0.0)	3 (0.0)		
ADI percentile, mean (SD)	39.46 (26.49)	42.45 (28.33)	38.30 (25.65)	<.001	Kruskal-Wallis (33.692; *df* = 1)

Abbreviations: ADI, area deprivation index; NA, not applicable.

^a^ Includes participants who did not or refused to provide an answer or indicated that they did not know.

^b^ Includes participants who indicated “Different,” “Gender queer,” or “Trans” for their gender.

### Wearable Device Data Collection

**Figure 1.**
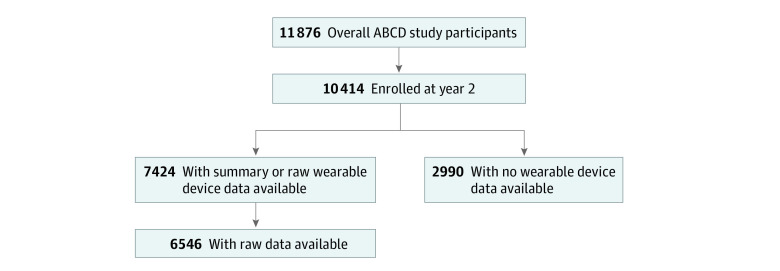
Flowchart of Wearable Device Data Availability From the Overall Adolescent Brain and Cognitive Development (ABCD) Study Cohort

Significant differences were seen between participants (7424 [71.3%]) who enrolled in the wearable device substudy (wearable device cohort [WDC]) compared with the 2990 (28.7%) who did not participate or share data (no wearable device cohort [NWDC]) (Figure 1 and Table 1). There was a significantly lower (−59%) relative proportion of Black children in the WDC (847 [11.4%]) than in the NWDC (577 [19.3%]; *P* < .001). In contrast, the WDC had a significantly higher (+132%) relative proportion of White children (4301 [57.9%]) than the NWDC (1314 [43.9%]; *P* < .001). Similar significant differences in relative proportion for Hispanic or Latino children were observed across the 2 cohorts (WDC, 1354 [18.2%]; NWDC, 694 [23.2%]; *P* < .001). Furthermore, a significantly lower proportion of children in the WDC (638 [8.6%]) were from households with income less than $24 999 compared with the NWDC (492 [16.5%]). Similarly, a notably lower proportion of parents whose children were in the WDC (1093 [14.7%]) had reached higher than an International Standard Classification of Education level of 3 (equivalent to grades 10-12 of high school) compared with the NWDC (705 [23.6%]). Table 1 and eAppendix 2.1 in Supplement 1 provide a complete comparison of differences in participant and parental demographic characteristics across WDC and NWDC.

### Participant Wearable Device–Based Retention

**Figure 2.**
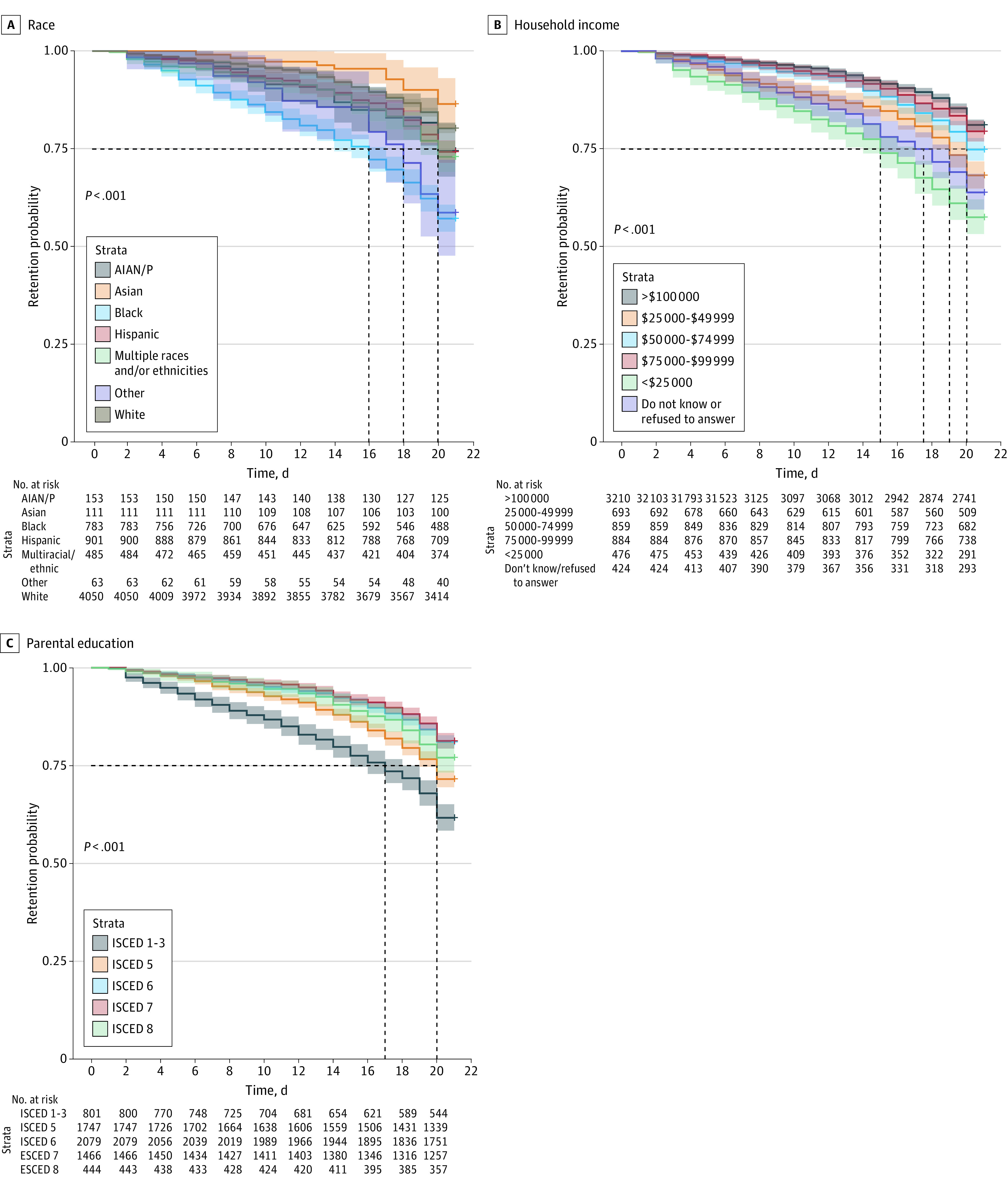
Kaplan-Meier Curves of Participant Retention Based on Device Wear in the 21-Day Observation Period, by Sociodemographic and Socioeconomic Factors Kaplan-Meier curves showed significant variation in participant retention based on device wear in the 21-day observation period by (A) participants’ race, (B) household income, and (C) parental education (based on International Standard Classification of Education [ISCED] levels). AIAN/P indicates American Indian, Alaska Native, and Pacific Islander.

Marked differences in participant retention (last day of device wear) were observed in the WDC subcohort that shared data (6546 participants). At 75% cohort retention, Black children shared their data for a significantly shorter duration (16 days, 95% CI, 14-17 days; *P* < .001) compared with children from other racial and ethnic groups (White children: 21 days; 95% CI, 21-21 days; *P* < .001; range across groups, 18-21 days) (Figure 2A). Parents’ SES varied notably between retention levels. Children living in households with incomes less than $25 000 had the lowest retention, at 15 days (95% CI, 14-17 days) (Figure 2B). Children whose parents completed education level was between ISCED 1 to 3 (at most, upper high school) were retained for a significantly shorter period (17 days; 95% CI, 15-18 days; *P* < .001) compared with children whose parents had completed an ISCED 5 level of education (associate’s degree equivalent in the United States) (20 days; 95% CI, 19-20 days). Sensitivity analysis showed similar differences in retention for participants recruited during the COVID-19 pandemic (eAppendix 2.3 in Supplement 1).

### Association Between Device Wear Time and Socioeconomic Indicators

**Figure 3.**
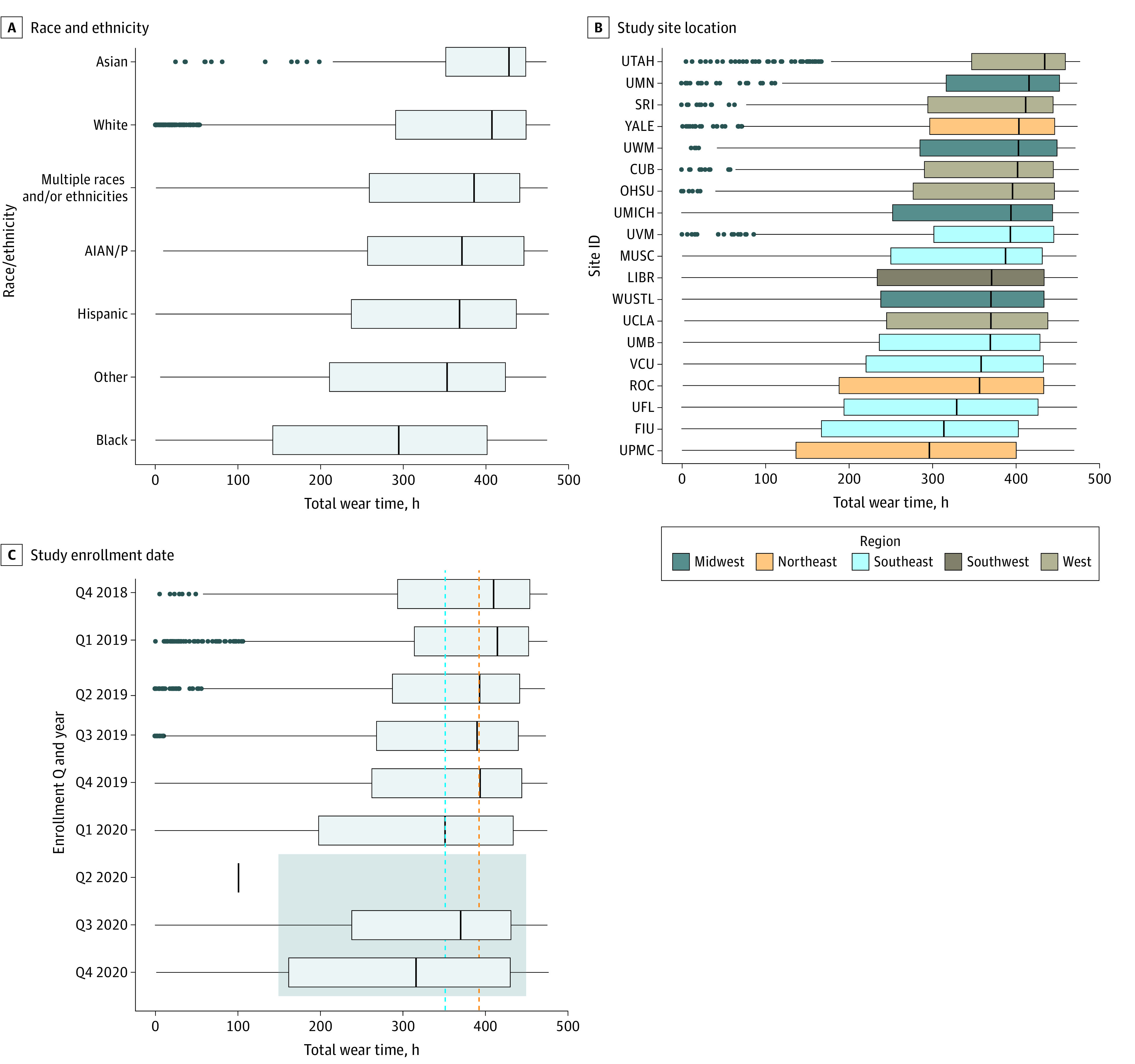
Bar Plots of Total Wear Time by Sociodemographic and Study-Related Factors Total wear time was by (A) race and ethnicity, (B) Adolescent Brain and Cognitive Development Study site location, and (C) participant enrollment period. C, Shading indicates months affected by the COVID-19 pandemic, with the dotted blue and orange lines showing the median wear time during and before the pandemic. CUB indicates University of Colorado Boulder; FIU, Florida International University; LIBR, Laureate Institute for Brain Research; MUSC, Medical University of South Carolina; OHSU, Oregon Health & Science University; Q, quarter; ROC, University of Rochester; SRI, SRI International; UCLA, University of California, Los Angeles; UFL, University of Florida; UMB, University of Maryland at Baltimore; UMICH, University of Michigan; UMN, University of Minnesota; UPMC, University of Pittsburgh; UTAH, University of Utah; UVM, University of Vermont; UWM, University of Wisconsin-Milwaukee; VCU, Virginia Commonwealth University; WUSTL, Washington University in St Louis; and YALE, Yale University.

We investigated factors associated with participants’ total device wear time during the observation period using a mixed-effects model. The median (IQR) total device wear time within the 20-day observation period was 400.7 hours (286.4-446.0 hours). However, participants from racial and ethnic minority groups wore their devices significantly less than their White counterparts (Black vs White children: β = −43.00 hours [95% CI, −55.11 to −30.88 hours]; *P* < .001) (Figure 3A and Table 2). Notably, study sites in the southwestern region of the United States had lower wear times (Southwest vs Midwest: β = −24.1 hours [95% CI: −43.77 to −4.43 hours]; *P* = .02), whereas sites in the western region of the US had the highest wear times (West vs Midwest: β = 4.07 hours [95% CI, −16.46 to 24.59 hours]; *P* < .001) (Figure 3B and Table 2). eAppendix 2.4 in Supplement 1 includes more details.

**Table 2.** Mixed-Effects Model Examining Confidence Intervals for Linear Mixed-Effect Model

Factor	Differential wear time (95% CI), h	*P* value
Fixed effects		
Intercept	361.36 (336.79 to 385.92)	<.001
Race/ethnicity		
White	1 [Reference]	NA
American Indian, Alaska Native, or Pacific Islander	−9.84 (−30.76 to 11.08)	.04
Asian	23.78 (−0.29 to 47.85)	.05
Black	−43.00 (−55.11 to −30.88)	<.001
Hispanic	−4.39 (−14.61 to 5.83)	.40
Multiple racial and/or ethnic groups	−1.99 (−13.97 to 9.99)	.75
Other	−22.61 (−53.30 to 8.08)	.15
Gender		
Male	1 [Reference]	NA
Different, gender queer, or trans	44.96 (−51.77 to 141.70)	.36
Do not know or refused to answer	29.80 (−106.79 to 166.40)	.67
Female	11.92 (5.85 to 17.99)	<.001
Weight category (BMI percentile)		
Healthy (5th to <85th)	1 [Reference]	NA
Missing	−9.95 (−30.39 to 10.49)	.34
Obesity (≥95th)	−8.97 (−17.80 to −0.15)	.046
Overweight (85th to ≤95th)	−9.35 (−18.16 to −0.54)	.04
Underweight (<5th)	1.64 (−14.10 to 17.37)	.84
Parental marital status		
Married	1 [Reference]	NA
Divorced, separated, widowed	−26.93 (−36.36 to −17.49)	<.001
Living with partner	−27.64 (−41.82 to −13.47)	<.001
Never married	−25.22 (−38.48 to −11.97)	<.001
Household income, $		
50 000-74 999	1 [Reference]	NA
<25 000	−25.51 (−40.73 to −10.28)	.001
25 000-49 999	−3.45 (−16.21 to 9.30)	.60
75 000-99 999	−5.49 (−17.35 to 6.36)	.36
>100 000	−11.04 (−21.43 to −0.65)	.04
Do not know	−8.80 (−29.00 to 11.41)	.39
Refused to answer	−29.89 (−48.66 to −11.13)	.002
Parental education		
ISCED 8	1 [Reference]	NA
ISCED 1-3	−29.55 (−45.95 to −13.15)	<.001
ISCED 5	−19.19 (−33.24 to −5.13)	.007
ISCED 6	−9.16 (−22.30 to 3.98)	.17
ISCED 7	−5.23 (−18.64 to 8.19)	.45
ADI quartile (percentiles)		
4th (75th to 100th)	1 [Reference]	NA
1st (1st to 24th)	9.67 (−3.68 to 23.03)	.16
2nd (25th to 49th)	16.38 (4.34 to 28.41)	.008
3rd (50th to 74th)	12.34 (0.27 to 24.41)	.045
Geographical region		
Midwest	1 [Reference]	NA
Northeast	−7.94 (−32.09 to 16.21)	.52
Southeast	−24.1 (−43.77 to −4.43)	.02
Southwest	−8.54 (−41.41 to 24.33)	.61
West	4.07 (−16.46 to 24.59)	.70
Random effects		
σ^2^	14456.37	NA
τ00 Site location	192.31	NA
τ00 Enrollment quarter	442.34	NA
ICC	0.04	NA
Site locations, No.	19	NA
Enrollment quarters, No.	9	NA
Total observations, No.	6114	NA
Marginal *R*^2^ (conditional *R*^2^)	0.070 (0.109)	NA

Abbreviations: ADI, area deprivation index; ICC, intraclass correlation; ISCED, International Standard Classification of Education; NA, not applicable.

In addition, device wear time was substantially lower for the population recruited during the COVID-19 pandemic than before the pandemic period (Figure 3C). eAppendix 2.2.2 in Supplement 1 includes more details regarding enrollment during the COVID-19 pandemic. We also observed 4 distinct longitudinal device wear patterns from the total daily wear time (eAppendix 2.5 in Supplement 1).

## Discussion

Our findings using more than 100 000 days of wearable data from one of the largest and most diverse wearable device deployments among more than 10 000 children nationwide show significant differences in data collection association with demographic and socioeconomic indicators. Specifically, (1) a significantly lower proportion of racial and ethnic minority youths enrolled into the wearable device substudy compared with White youths; (2) racial and ethnic minority youths, in particular Black children, and those from lower SES households, shared data for fewer days (ie, lower retention in the 21-day observation period) and had significantly less total wear time compared with White children or those from higher SES households; (3) wear time was associated with external factors, such as recruitment site; and (4) the COVID-19 pandemic was associated with device wear time. As consumer-grade wearable devices become more common in health research, these data-driven findings could help to guide the development of strategies for reaching, recruiting, and retaining participants from racial and ethnic minority groups and lower SES populations.

It is well known that racial and ethnic minority participants are underrepresented in clinical trials.^49^ Our analyses reflected this underrepresentation, with the relative proportion of the total Black children (59% lower WDC vs NWDC) enrolled having wearable device data available being significantly lower than that of White children (132% higher in WDC vs NWDC) (Table 1). As the use of smart devices widens in health research and care, there is an urgent need to address known technical,^30^ structural, and systemic barriers^27^ to enable equitable and representative use of technology for health data collection. Structural racism is also deeply rooted in societal structures that control power and resources, leading to discriminatory and inequitable systems that can reinforce discriminatory beliefs and values. The inequitable allocation of resources (eg, the education system)^50^ could be due to multiple factors, including reduced neighborhood safety; access to green space, health care, and education; and diminished opportunities for upward mobility, resulting in individuals residing in disadvantaged neighborhoods for longer.^32^ These factors can negatively affect physical activity levels, particularly for Black communities, which have historically lower trust in health care and research^51,52^ due to historical discrimination from significant events such as Sims, Lacks, and Tuskegee.^53^

There was also poorer retention of Black compared with White families, which likely contributed to lower overall wear time. Future wearable device research should address various sociotechnical and human factors to improve wearable device–based data collection. These can range from participants’ and their families’ understanding of the study, consent and assent language, the potential influence of personal data sharing, its secondary usage, and their overall trust in the study team.^32^ Characteristics of the study team may also inform whether participants opt in to studies, as racial and ethnic minority communities are more likely to trust those that are like them and understand their experiences.^54,55,56,57^ Failure to do so may result in findings that reinforce negative stereotypes in racial and ethnic minority youths,^58^ becoming more detrimental than beneficial, specifically for their communities. Finally, the present wearable technology is known to be less reliable for people with darker skin tones and higher body mass index (eg, inaccurate HR measurement).^30^ The interplay of many such sociotechnical factors can lead to nonuniform participant engagement and data collection among racial and ethnic minority populations, which may result in the learning of treatment paradigms that are not appropriate or inadequate for certain underrepresented populations and may further contribute to health disparities in disease and treatment outcomes and access to care.

Despite some of the above challenges, wearable devices may still provide an avenue for large-scale health monitoring in real-world settings guiding early detection and intervention opportunities. Personalized just-in-time adaptive interventions^59^ (JITAIs) are one promising digital approach that processes in-the-moment wearable data and provides timely and appropriate interventions when support is most needed. JITAIs may improve the effectiveness of evidence-based interventions by identifying vulnerable states with passive measures of treatment targets (eg, sleep, physical activity) and providing in-the-moment intervention prompts to affect proximal treatment targets and improve distal outcomes (eg, psychopathology symptoms). These have been used to treat a variety of adolescent health issues^60,61,62,63,64^ effectively. While less studied, JITAIs in the context of mental health care is an area of interest identified by the National Institute of Mental Health^63^ and holds promise for changing the landscape of child and adolescent mental health care. However, as evidenced in the present data set, there is an urgent need to understand and address SDoH factors, particularly for racial and ethnic minority communities or those with lower SES, such that they are recruited and retained equitably to their White counterparts.

### Limitations

The analysis of retention and wear time of wearable devices should be viewed in the context of certain limitations linked to the deployment of these devices. First, our approximation of wear time was based on available per-minute HR values, which have been used in previous studies^22^; however, wearable device data are affected by motion artifacts.^64,65^ Thus, our measure of wear time may be an imperfect proxy. The HR sensor can also be affected by skin tone, which may bias wearable devices’ data against racial and ethnic minority youths who may have darker skin tones.^64^ In addition, racial and ethnic minority groups are disproportionately affected by diabetes and obesity,^66^ which can affect HR measurement.^67^ Other variables, such as gender affecting cutaneous blood flow,^68^ may also affect data collected from wearable devices. Further research is needed to assess wear time accurately by fusing multimodal sensor data. Second, the wearable devices used in this study were not certified to be worn by those younger than 13 years, and most participants were 11 to 12 years of age at the time of data collection. While more research is necessary to understand how these devices perform with younger children, they have been found to have adequate performance.^20^ Third, nearly one-third (28.7%) of the Y2 cohort did not have wearable device data (eAppendix 2.6.2 in Supplement 1); it was unclear how many opted out or were unable to participate in the substudy due to factors, such as participants not being offered a wearable device due to site-specific availability or not being mailed a device for remote assessment due to the COVID-19 pandemic. The latter may have affected wear time, especially for racial and ethnic minority youths who may have been disproportionately affected by psychosocial, economic, and health issues, thus having a diminished capacity to engage with the ABCD Study.

## Conclusions

In one of the largest deployments of wearable devices in children, we found evidence that children from lower SES backgrounds and Black children were less likely to participate, share data, and engage in wearable device–based health research than those with higher SES backgrounds and White children. With the use of wearable devices being an emerging area of health research, it is critical to understand and address factors that can significantly affect wearable device data collection for robust and generalizable evidence generation.

## Notes

Supplement 1. **eAppendix 1.** Supplementary Methods **eAppendix 2.** Supplementary Results
eReferences.

Supplement 2.
Data Sharing Statement
