# Supplementary material for: Association of Demographic and Socioeconomic Indicators With the Use of Wearable Devices Among Children
Source: JAMA Netw Open. 2023 Mar 30;6(3):e235681. doi: 10.1001/jamanetworkopen.2023.5681 (PMC10064258; doi:10.1001/jamanetworkopen.2023.5681)
Supplement: Supplement 1. — eAppendix 1. Supplementary Methods eAppendix 2. Supplementary Results eReferences. [file jamanetwopen-e235681-s001.pdf]

Supplemental Online Content

Kim EH, Jenness JL, Miller AB, et al. Association of demographic and socioeconomic indicators with the use of wearable devices among children. *JAMA Netw Open.* 2023;6(3):e235681. doi:10.1001/jamanetworkopen.2023.5681

eAppendix 1. Supplementary Methods

eAppendix 2. Supplementary Results

eReferences.

This supplemental material has been provided by the authors to give readers additional information about their work.

© 2023 Kim EH et al. *JAMA Network Open.*

eAppendix 1. Supplementary Methods

eAppendix 1.1. Secondary sociodemographic variables

ADI: The national percentile is based upon the neighborhood's ADI value, binned to the nearest 1%, where 1% indicates the lowest level of disadvantage, whereas 100% indicates the highest level of disadvantage.

Race/ethnicity: Both participants' and parents' race/ethnicity was compressed for their original values into 8 larger categories. Participants who indicated more than one value was grouped as "Multiracial/ethnic" and any Pacific Islanders, Alaskan Natives or (Native) American Indians were grouped as AIAN/P. Those of all Asian race/ethnicities were grouped into "Asian." Finally, if any indicated that they were of Hispanic/Latin ethnicity, they were grouped as "Hispanic" regardless of if they chose other ethnicities in the demographic survey.

BMI: Using the children's weight and height measurements taken at the respective ABCD Study timepoints, we calculated their BMI (kg/m^2) and converted it to a z-score (BMI_z) using the CDC 2000 Growth Chart values[1]. BMI_z scores were categorized into underweight (< 5th percentile), healthy (> 5th percentile and < 85th percentile), overweight (> 85th percentile), and obese (> 95th percentile).

Education: Parental education values (originally 21 options) were re-coded into 8 categories per the International Standard Classification of Education (ISCED)[2], ranging from 1 to 8 being the equivalent of primary school and doctoral degrees in the U.S., respectively.

Household income: Combined family income (referred to as "household income") levels were compressed from the original 10 values to 5 levels using this conversion table below:

Original values	New values
< $5000	< $24,999

© 2023 Kim EH et al. *JAMA Network Open.*

$5000 - $11,999	
$12,000 - $15,999	
$16,000 - $24,999	
$25,000 - $34,999	$25,000 - $49,999
$35,000 - $49,999	
$50,000 - $74,999	$50,000 - $74,999
$75,000 - $99,999	$75,000 - $99,999
$100,000 - $199,999	> $100,000
> $200,000	
Don’t know	Don’t know/Refused to answer
Refused to answer	

eAppendix 1.2. Wearable device-based data collection

Research staff at each site assisted parents in setting up the wearable device (Fitbit) and its companion mobile application (app), including turning off app notification and geolocation capabilities and instructing how to charge and sync the device. To collect the data, the Fitbit app was downloaded to either the child’s or parent’s cellular device, to which the wearable device would sync through Bluetooth and transmit their data upon close proximity. To protect participants’ privacy, the study team created de-identified Fitbit accounts to connect the devices to the Fitbit app. The phone app then securely streams the data via wireless technology to Fitbit’s proprietary secure cloud storage. Software developed by Fitabase Inc.[3] was used to then retrieve the data from Fitbit’s servers, and from there, data were securely imported to ABCD study servers. The ABCD Study team monitored incoming wearable data and contacted families if their child’s wearable device data had not been uploaded for 4 days. Upon completion of the 3 week observation period, families were instructed to return the device by mail. Participants were sent a gift card for participation. The data was made available in two formats:

© 2023 Kim EH et al. *JAMA Network Open.*

the “Daily Summary” format which summarized participants’ metrics (physical activity, sleep, etc) per day, and the high-resolution per min metrics (hereon referred to as “raw” data).

eAppendix 1.3. Retention analysis

To assess the joint effect of multiple variables of interest on participants’ retention in the study, we initially used a multivariate Cox proportional hazard (Cox-PH) model[4]. However the proportional hazard assumptions of Cox-PH model tested using the Schoenfeld individual test were not met. As a result, we used a univariate non-parametric approach using Kaplan-Meier (KM)[5] curves to assess retention. The test for statistical significant differences between KM curves was done using stratified log-rank test. We used two approaches to evaluate participant retention, for which both used data from all 6,546 children with available wearable device data. The first was no censoring, where if the last active day recorded by a participant’s wearable device was between the observation period of 21 days, we considered the participant to have stopped using their wearable device. The second approach taken was right-censoring for determining the sensitivity of our first approach where we extended our observation period to 35 days. Thus, if a participant had initially been considered as no longer active” in our first approach but had recorded wearable device activity in the extended observation period, they would be re-coded as “active” and considered right-censored.

© 2023 Kim EH et al. *JAMA Network Open.*

eAppendix 2. Supplementary Results

eAppendix 2.1. ABCD Study Parental Demographics

	Overall	Wearable Device Availability		*P value*
		No Wearable Device Cohort (NWDC)	Wearable Device Cohort (WDC)	
**n**	10414	2990	7424	
**Age (mean (SD))**	43.13 (28.56)	43.85 (40.31)	42.83 (22.12)	0.102
**Race/Ethnicity (%)**				<0.001
White	6341 (60.9)	1501 (50.2)	4840 (65.2)	
Hispanic	1719 (16.5)	603 (20.2)	1116 (15.0)	
Black	1406 (13.5)	568 (19.0)	838 (11.3)	
Asian	326 (3.1)	115 (3.8)	211 (2.8)	
Multiracial/ethnic	287 (2.8)	99 (3.3)	188 (2.5)	
AIAN/P	188 (1.8)	48 (1.6)	140 (1.9)	
Other	147 (1.4)	56 (1.9)	91 (1.2)	
**Gender (%)**				0.344
Female	9277 (89.1)	2648 (88.6)	6629 (89.3)	
Male	1111 (10.7)	332 (11.1)	779 (10.5)	
Different/Gender Queer/Trans	21 (0.2)	9 (0.3)	12 (0.2)	
Don't know/Refused to answer	1 (0.0)	0 (0.0)	1 (0.0)	
**Education (%)**				<0.001
ISCED 6	3038 (29.2)	769 (25.7)	2269 (30.6)	
ISCED 5	2763 (26.5)	781 (26.1)	1982 (26.7)	
ISCED 7	2103 (20.2)	512 (17.1)	1591 (21.4)	
ISCED 1-3	1798 (17.3)	705 (23.6)	1093 (14.7)	
ISCED 8	696 (6.7)	217 (7.3)	479 (6.5)	
Refused to answer	16 (0.2)	6 (0.2)	10 (0.1)	
**Household Income (%)**				<0.001
< $24,999	1130 (10.9)	492 (16.5)	638 (8.6)	
$25,000 - $49,999	1219 (11.7)	357 (11.9)	862 (11.6)	
$50,000 - $74,999	1244 (11.9)	285 (9.5)	959 (12.9)	

© 2023 Kim EH et al. *JAMA Network Open.*

	Overall	Wearable Device Availability		
		No Wearable Device Cohort (NWDC)	Wearable Device Cohort (WDC)	*P value*
$75,000 - $99,999	1333 (12.8)	363 (12.1)	970 (13.1)	
> $100,000	4599 (44.2)	1108 (37.1)	3491 (47.0)	
Don't know/Refused to answer	889 (8.5)	385 (12.9)	504 (6.8)	
**Employment Status (%)**				<0.001
Working now, FULL TIME/PART TIME	7678 (75.5)	2009 (69.2)	5669 (77.9)	
Stay at home parent	1465 (14.4)	450 (15.5)	1015 (14.0)	
Looking for work	345 (3.4)	136 (4.7)	209 (2.9)	
**Employment Status (%)**				<0.001
Disabled: permanently or temporarily	206 (2.0)	76 (2.6)	130 (1.8)	
Temporarily laid off	150 (1.5)	96 (3.3)	54 (0.7)	
Student	143 (1.4)	50 (1.7)	93 (1.3)	
Unemployed, not looking for work	107 (1.1)	56 (1.9)	51 (0.7)	
Retired	78 (0.8)	25 (0.9)	53 (0.7)	
Other/Refused to answer	4 (0.0)	4 (0.1)	0 (0.0)	
**Marital Status (%)**				<0.001
Married	7055 (67.7)	1840 (61.5)	5215 (70.2)	
Never married	1076 (10.3)	421 (14.1)	655 (8.8)	
Divorced	1035 (9.9)	284 (9.5)	751 (10.1)	
Living with partner	671 (6.4)	235 (7.9)	436 (5.9)	
Separated	395 (3.8)	131 (4.4)	264 (3.6)	
Widowed	111 (1.1)	45 (1.5)	66 (0.9)	
Refused to answer	71 (0.7)	34 (1.1)	37 (0.5)	

© 2023 Kim EH et al. *JAMA Network Open.*

eAppendix 2.2. Differential enrollment of wearable device sub-study participants

eAppendix 2.2.1. Enrolment of wearable device sub-study participants by quarter-year

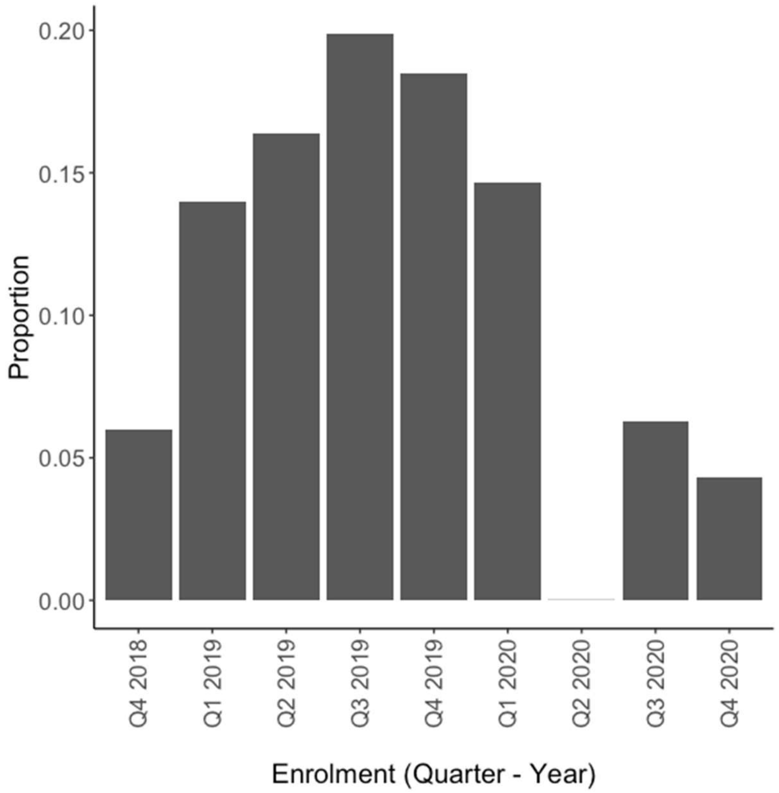

© 2023 Kim EH et al. *JAMA Network Open.*

eAppendix 2.2.2. Comparison of participants' demographics based on the recruitment period - enrolled before pre-COVID19 or during COVID-19 pandemic

	Recruited during the COVID-19 pandemic		
	No	Yes	*P* value
**n**	5852	694	
**Total wear-time (mean (SD))**	20562.02 (7630.70)	18691.34 (8228.06)	<0.001
**Child race/ethnicity (%)**			0.199
White	3639 (62.2)	411 (59.2)	
Hispanic	808 (13.8)	93 (13.4)	
Black	688 (11.8)	95 (13.7)	
Multiracial/ethnic	422 (7.2)	63 (9.1)	
AIAN/P	134 (2.3)	19 (2.7)	
Asian	104 (1.8)	7 (1.0)	
Other	57 (1.0)	6 (0.9)	
**Child gender (%)**			0.899
Male	3036 (51.9)	354 (51.0)	
Female	2804 (47.9)	339 (48.8)	
Different/Gender Queer/Trans	7 (0.1)	1 (0.1)	
Don't know/Refused to answer	3 (0.1)	0 (0.0)	
**ADI percentile (mean (SD))**	39.36 (25.56)	41.81 (27.76)	0.026
**Weight category (%)**			<0.001
Healthy	3744 (64.0)	297 (42.8)	
Obesity	969 (16.6)	91 (13.1)	
Missing	8 (0.1)	217 (31.3)	
Overweight	896 (15.3)	73 (10.5)	
Underweight	235 (4.0)	16 (2.3)	
**Site Region (%)**			<0.001
Midwest	1493 (25.5)	115 (16.6)	
Northeast	658 (11.2)	87 (12.5)	
Southeast	1556 (26.6)	142 (20.5)	
Southwest	500 (8.5)	103 (14.8)	
West	1645 (28.1)	247 (35.6)	

© 2023 Kim EH et al. *JAMA Network Open.*

**Education (%)**			0.412
ISCED 1-3	725 (12.4)	76 (11.0)	
ISCED 5	1564 (26.7)	183 (26.4)	
	During the COVID-19 pandemic		
	No	Yes	*P* value
**Education (%)**			0.412
ISCED 6	1865 (31.9)	214 (30.8)	
ISCED 7	1289 (22.0)	177 (25.5)	
ISCED 8	401 (6.9)	43 (6.2)	
Refused to answer	8 (0.1)	1 (0.1)	
**Household income (%)**			0.63
< $24,999	201 (3.6)	24 (3.6)	
$25,000 - $49,999	608 (10.8)	85 (12.6)	
$50,000 - $74,999	769 (13.7)	90 (13.3)	
$75,000 - $99,999	800 (14.2)	84 (12.4)	
> $100,000	2874 (51.1)	336 (49.7)	
Don't know/Refused to answer	367 (6.5)	57 (8.4)	
**Employment (%)**			<0.001
Working now, FULL TIME/PART TIME	4580 (78.7)	511 (73.8)	
Stay at home parent	759 (13.0)	96 (13.9)	
Looking for work	146 (2.5)	21 (3.0)	
Disabled: permanently or temporarily	105 (1.8)	15 (2.2)	
Temporarily laid off	24 (0.4)	15 (2.2)	
Student	63 (1.1)	14 (2.0)	
Unemployed, not looking for work	32 (0.5)	5 (0.7)	
Retired	35 (0.6)	7 (1.0)	
Other/Refused to answer	78 (1.3)	8 (1.2)	
**Marital status (%)**			0.248
Married	4196 (71.7)	479 (69.0)	
Never married	503 (8.6)	63 (9.1)	
Divorced	576 (9.8)	88 (12.7)	
Living with partner	320 (5.5)	33 (4.8)	
Separated	180 (3.1)	25 (3.6)	

Widowed	54 (0.9)	4 (0.6)	
Refused to answer	23 (0.4)	2 (0.3)	

© 2023 Kim EH et al. *JAMA Network Open.*

eAppendix 2.3. Retention

eAppendix 2.3.1 Number of days the 75% of the cohort is retained stratified by cohort socio-demographic/economic factors

	Participant enrollment		
	Overall (n = 6,546)	Pre-COVID (2019 - Mar. 2020) (n = 5,852)	COVID (Mar. 2020 -) (n = 694)
**Strata**	75% Retention (95% CI)		
**Race/Ethnicity**			
AIAN/P	20 (20 - NR)	NR (20 - NR)	14 (12 - NR)
Asian	NR (NR - NR)	NR (NR - NR)	NR (8 - NR)
Black	16 (14 - 17)	16 (15 - 17)	12 (11 - 18)
Hispanic	20 (20 - NR)	NR (20 - NR)	15 (11 - NR)
Multiracial/ethnic	20 (19 - NR)	NR (20 - NR)	13 (9 - 19)
White	NR (NR - NR)	NR (NR - NR)	18 (16 - 20)
Other	18 (16 - 20)	18 (16 - 20)	16 (5 - NR)
**Weight Category**			
Healthy	NR (NR - NR)	NR (NR - NR)	16 (13 - 19)
Missing	18 (15 - 20)	13.5 (5 - NR)	19 (15 - NR)
Obesity	19 (19 - 20)	20 (19 - 20)	14 (12 - 18)
Overweight	20 (20 - NR)	20 (20 - NR)	17 (13 - NR)
Underweight	NR (NR - NR)	NR (NR - NR)	NR (14 - NR)
**Gender**			
Female	NR (NR - NR)	NR (NR - NR)	17 (15 - 19)
Male	NR (20 - NR)	NR (NR - NR)	17 (13 - 19)
**Parental Education**			
ISCED 1-3	17 (15 - 18)	18 (16 - 19)	11 (6 - 13)
ISCED 5	20 (19 - 20)	20 (20 - NR)	15 (13 - 19)
ISCED 6	NR (NR - NR)	NR (NR - NR)	19 (16 - 20)
ISCED 7	NR (NR - NR)	NR (NR - NR)	20 (18 - NR)
ISCED 8	NR (20 - NR)	NR (NR - NR)	14 (9 - 19)

© 2023 Kim EH et al. *JAMA Network Open.*

	Overall (n = 6,546)	Pre-COVID (2019 - Mar. 2020) (n = 5,852)	COVID (Mar. 2020 -) (n = 694)
**Parental Employment**			
Disabled	14 (12 - 18)	16 (13 - 19)	11 (7 - 13)
Laid off/Unemployed/Retired	16 (10 - 19)	19 (15 - NR)	10 (8 - 16)

	Participant enrollment		
	Overall (n = 6,546)	Pre-COVID (2019 - Mar. 2020) (n = 5,852)	COVID (Mar. 2020 -) (n = 694)
**Parental Employment**			
Looking for work	18 (15 - NR)	20 (16 - NR)	12 (3 - NR)
Maternity leave	NR (14 - NR)	NR (14 - NR)	NR (NA - NA)**
Sick leave	NR (NR - NR)	NR (NR - NR)	NR
**Parental Employment**			
Stay at home parent	NR (NR - NR)	NR (NR - NR)	20.5 (16 - NR)
Student	20 (19 - NR)	NR (19 - NR)	19 (11 - NR)
Working now	NR (NR - NR)	NR (NR - NR)	17 (15 - 19)
Refused to answer	17 (14 - NR)	17 (14 - NR)	20 (NA - NA)**
Other	20 (18 - NR)	NR (18 - NR)	12.5 (5 - NR)
**Marital status**			
Divorced/Separated/Widowed	19 (18 - 20)	20 (19 - 20)	13 (10 - 19)
Living with partner	19 (17 - 20)	19 (17 - 20)	13 (8 - 19)
Married	NR (NR - NR)	NR (NR - NR)	19 (17 - 20)
Never married	16 (15 - 17)	17 (16 - 18)	11 (8 - 15)
**Household income**			
< $25,000	15 (14 - 17)	16 (15 - 17)	11 (7 - 13)
$25,000 - $49,999	19 (18 - 20)	20 (19 - 20)	11 (7 - 16)
$50,000 - $74,999	20 (20 - NR)	NR (20 - NR)	18 (14 - NR)
$75,000 - $99,999	NR (NR - NR)	NR (NR - NR)	19 (15 - NR)
> $100,000	NR (NR - NR)	NR (NR - NR)	20 (19 - NR)
Don't know/Refused to answer	17.5 (15 - 19)	18 (17 - 20)	12 (10 - 16)

NR: Not reached 75% retention. This indicates that 75% of cohort was retained for the complete observation period i.e., 21 days.
*** indicates a sample size of n = 1,* mainly due to data sparsity in a smaller sub-sample

© 2023 Kim EH et al. *JAMA Network Open.*

eAppendix 2.3.2. Test statistics from Schoenfeld test to assess CoxPH model assumptions

	Chi-square statistic	Degrees of freedom	*P* value
Race/Ethnicity	11.00	6	0.08850
Gender	5.92	1	0.01496
ADI	12.920	1	0.00033
Parental Education	8.04	4	0.08997
Household income	16.08	5	0.00661
Global	36.43	17	0.00401

© 2023 Kim EH et al. *JAMA Network Open.*

eAppendix 2.3.3. Results of stratified log-rank test for participants' retention in the study based on the last day of wearable device wear

Sociodemographic factors	n	observed	expected	Chi-square value
**Race/Ethnicity**				
AIAN/P	153	39	36.64475912	0.1513766156
Asian	111	15	28.62306308	6.483856994
Black	783	335	162.750056	182.3043504
Hispanic	901	232	215.6226687	1.24391829
Multiracial/ethnic	485	131	114.5980582	2.347541468
Other	63	26	13.91618442	10.49271803
White	4050	799	1004.845211	42.16793816
**Weight category**				
Healthy	4041	884	989.0533538	11.1583537
Missing	225	71	49.77112401	9.054751818
Obesity	1060	328	244.9081205	28.19122704
Overweight	969	250	230.7408799	1.607490218
Underweight	251	44	62.52652172	5.489382706
**Gender**				
Female	3143	737	762.8888188	0.8785434015
Male	3390	835	809.1111812	0.8283545616
**Parental Education**				
ISCED 1-3	801	307	173.0047372	103.7817272
ISCED 5	1747	497	410.8846024	18.04852667
ISCED 6	2079	394	515.2803107	28.54546052
ISCED 7	1466	274	366.9646454	23.55111152
ISCED 8	444	102	107.8657043	0.3189752227
**Employment status**				
Disabled	120	54	24.34465347	36.12454698
Laid off/ Unemployed/Retired	118	45	24.71970642	16.63815502
Looking for work	167	50	37.17953944	4.420824237
Maternity leave	9	1	2.288283585	0.7252923573
Other	86	22	20.28642337	0.1447443349

© 2023 Kim EH et al. *JAMA Network Open.*

Refused to answer	15	5	3.471623315	0.6728654227
Sick leave	8	0	2.178426437	2.178426437
Stay at home parent	855	176	211.1845554	5.861948263
**Sociodemographic factors**	n	observed	expected	Chi-square value
Student	77	20	18.54434655	0.1142626922
Working now	5091	1204	1232.802442	0.6729226341
**Marital status**				
Divorced/Separated/ Widowed	927	284	210.5022249	25.66207055
Living with partner	353	121	79.1537471	22.12288042
Married	4675	916	1159.073757	50.97592008
Never married	566	246	118.2702713	137.9457695
**Household income**				
< $25,000	476	202	97.75773833	111.1569202
> $100,000	3210	605	802.797831	48.7345387
$25,000 - $49,999	693	220	158.9991027	23.40333634
$50,000 - $74,999	859	216	206.7858537	0.410572053
$75,000 - $99,999	884	181	217.5738341	6.14800648
Don't know/Refused to answer	424	153	93.08564018	38.56374094

© 2023 Kim EH et al. *JAMA Network Open.*

eAppendix 2.4. Linear mixed-effect modeling

Marital status was also found to be significantly linked ($P < .001$) to wearable device wear-time ($\boldsymbol{\beta}_{\text{divorced/separated/widowed}}$ = -26.93 hours [95% CI: -36.36 – -17.49]; $\boldsymbol{\beta}_{\text{Living with a partner}}$ = -27.64 hours [95% CI: -41.82 – -13.47]) when compared to parents who were married. Children from households with a lower socio-economic status had significantly ($P < .001$) lower wearable device wear-time. For example, children whose household income was lower than < $25,000 wore the devices for less time ($\boldsymbol{\beta}$ = -25.51 hours; [95% CI: -40.73 – -10.28]) compared to those with household income between $50,000 - $74,999. Gender also had a significant ($P < .001$) effect, where female children had higher total wear-time ($\boldsymbol{\beta}_{\text{Females}}$ = 11.92 hours [95% CI: 5.85 – 17.99]). Finally, compared to devices from children whose parents had completed or reached the highest level of education (ISCED 8; doctorate degree equivalent), devices from children whose parents had reached an ISCED level of 5 (associate degree equivalent) or below had significantly lower recorded wear-time ($\boldsymbol{\beta}_{\text{ISCED 1-3}}$ = -29.55 hours [95% CI: -45.95 – -13.15]; $P < .001$, $\boldsymbol{\beta}_{\text{ISCED 5}}$ = -19.19 hours [95% CI: -33.24 – -5.13]; $P = .007$).

© 2023 Kim EH et al. *JAMA Network Open.*

eAppendix 2.5. Longitudinal wearable device wear-time analysis

eAppendix 2.5.1. Methods

To examine patterns of longitudinal wearable device wear patterns, we performed unsupervised K-means clustering using the total wear-time per day for each participant, calculated by taking the sum of the total number of minutes with HR values. The first day of wear within the 21-day observation period was excluded due to high variance in wear start times, resulting in 20 days' of daily wear-time by using HR as the proxy for 6,424 participants. The clustering was performed using the Manhattan distance to account for high dimensionality of the wear-time data. We determined the appropriate number of clusters to be 4 clusters to group the participants by using the elbow method and examining the resulting elbow plot (eAppendix 2.5.3). Longitudinal wearable device wear patterns were determined by performing unsupervised *K*-means clustering using the total wear-time per day for each participant, using the Manhattan distance to account for the high dimensionality of the wear-time data. We used the elbow method to select the appropriate number of clusters.

eAppendix 2.5.2. Results

Participants' daily wearable device wear-time showed four distinct patterns (eAppendix 2.5.4). The majority of the participants (65%; *n* = 4,264) that grouped in cluster 1 (C1) had high wear-time (IQR): (median: 419.80 [43.08] hours; 87% wear-time during the observation period). The rest of the 2,181 (35%) participants in clusters 2-4 had significantly lower wear-time. Cluster 2 with 798 (12.2%) participants had moderate wear-time (271.27 [45.47] hours; 56.5% during the observation period) whereas Cluster 3 with 974 (14.9%) participants had significantly lower wear-time (119.70 [62.01] hours; 20.8% wear-time). Notably, Cluster 4 with 510 (7.8%) participants showed moderate wear after inactivity during the first week of the study (231.48 [59.73] hours; 48.3% wear-time).

Statistically significant differences (P < .001) in participant demographics were seen across the four wear-time-based clusters (C1-4) (eAppendix 2.5.5). White participants were notably overrepresented by

21.6% in the largest and highest wear-time cluster (C1: 66.7%, C3: 45.1%), whereas Black participants were overrepresented by 17.9% in the lowest wear-time cluster (C1: 8.2%, C3: 26.1%). Children from lower-income households (< $49,999) were overrepresented by 16.8% in the lowest wear-time cluster (C1: 14.2%, C3: 31.0%) whereas those from higher-income households ($100,000) were overrepresented by 12.3% in the highest wear-time cluster (C1: 37.7%, C3: 25.4%) (eAppendix 2.5.5).

eAppendix 2.5.3. Elbow plot for unsupervised clustering of longitudinal wearable device wear time patterns

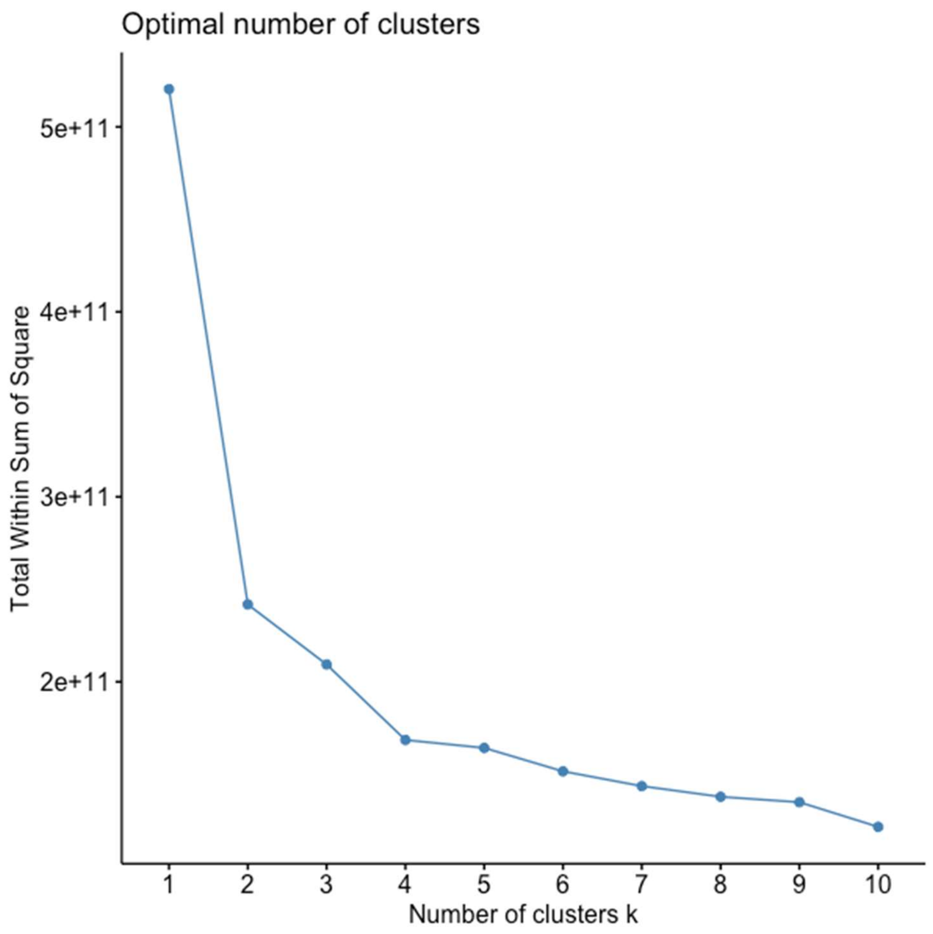

© 2023 Kim EH et al. *JAMA Network Open.*

eAppendix 2.5.4. Heatmap showing clustering of participants based on daily wearable device wear-time patterns during the study observation period

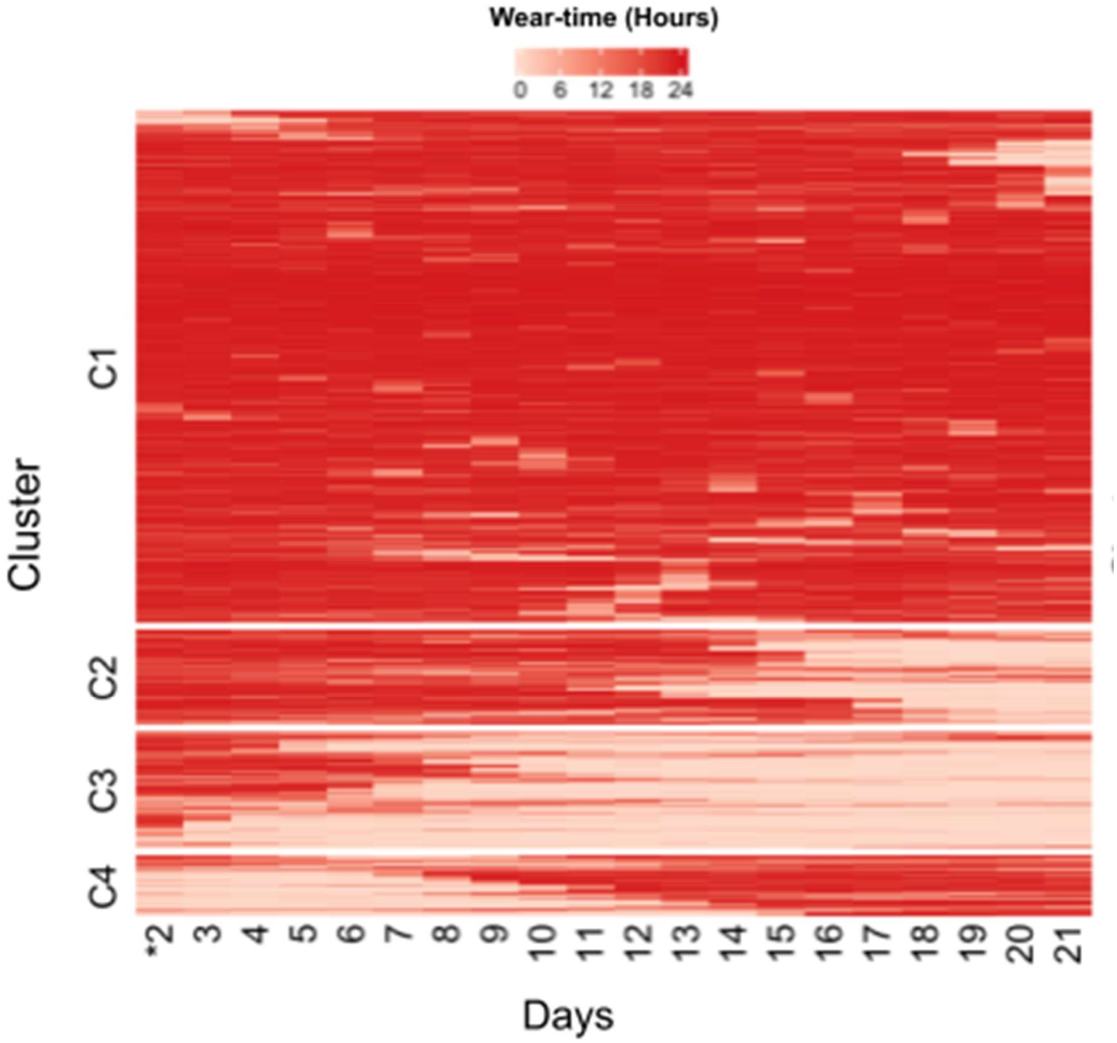

Heatmap showing distinct clusters (C1-4) of participants' longitudinal wearable device wear-time over the study observation period. Cluster 1 had the majority of participants with the highest median wear-time (419. 8 hours; 87% of the observation period*) followed by clusters C2-4 with wear-time ranging between 20.8 - 56.5%. (*The first day of wear was excluded to normalize wear-time)*.

© 2023 Kim EH et al. *JAMA Network Open.*

eAppendix 2.5.5. Comparison of participant demographics across four clusters based on longitudinal wearable device wear time

	C1	C2	C3	C4	*P* value
**n**	4264	798	974	510	
Total wear-time (mean (SD))	419.80 (43.08)	271.27 (45.47)	99.72 (62.01)	231.48 (59.73)	<0.001
**Age (mean (SD))**	11.96 (0.71)	11.98 (0.73)	11.98 (0.71)	11.98 (0.71)	0.742
**Race/Ethnicity (%)**					<0.001
White	2844 (66.7)	459 (57.5)	439 (45.1)	308 (60.4)	
Hispanic	547 (12.8)	105 (13.2)	166 (17.0)	83 (16.3)	
Black	349 (8.2)	118 (14.8)	254 (26.1)	62 (12.2)	
Multiracial/ethnic	305 (7.2)	76 (9.5)	67 (6.9)	37 (7.3)	
AIAN/P	94 (2.2)	23 (2.9)	23 (2.4)	13 (2.5)	
Asian	88 (2.1)	8 (1.0)	11 (1.1)	4 (0.8)	
Other	37 (0.9)	9 (1.1)	14 (1.4)	3 (0.6)	
**Gender (%)**					0.029
Male	2171 (50.9)	398 (49.9)	549 (56.4)	272 (53.3)	
Female	2086 (48.9)	395 (49.6)	424 (43.6)	238 (46.7)	
Different/Gender Queer/Trans	5 (0.1)	3 (0.4)	0 (0.0)	0 (0.0)	
Don't know/Refused to answer	2 (0.0)	1 (0.1)	0 (0.0)	0 (0.0)	
**BMI category (%)**					<0.001
Healthy	2746 (64.4)	473 (59.3)	514 (52.8)	308 (60.4)	
Obesity	611 (14.3)	144 (18.0)	219 (22.5)	86 (16.9)	
Overweight	598 (14.0)	131 (16.4)	173 (17.8)	67 (13.1)	
Underweight	179 (4.2)	27 (3.4)	27 (2.8)	18 (3.5)	
Missing	130 (3.0)	23 (2.9)	41 (4.2)	31 (6.1)	

© 2023 Kim EH et al. *JAMA Network Open.*

eAppendix 2.6: ABCD Study cohort demographic details at Year 2 follow up

eAppendix 2.6.1. Site-specific recruitment of Y2 ABCD Study cohort

Site ID	State	Count	Proportion
UTAH	Salt Lake City, Utah	940	9.03%
LIBR	Tulsa, Oklahoma	723	6.94%
UCSD	San Diego, California	687	6.60%
WUSTL	St. Louis, Missouri	652	6.26%
UMICH	Ann Arbor, Michigan	627	6.02%
YALE	new Haven, Connecticut	582	5.59%
OHSU	Portland, Oregon	556	5.34%
FIU	Miami, Florida	549	5.27%
CUB	Boulder, Colorado	531	5.10%
UVM	Burlington, Vermont	520	4.99%
UMB	Baltimore, Maryland	512	4.92%
VCU	Richmond, Virginia	511	4.91%
UMN	Minneapolis, Minnesota	490	4.71%
UPMC	Pittsburgh, Pennsylvania	380	3.65%
UCLA	Los Angeles, California	359	3.45%
MUSC	Charleston, South Carolina	349	3.35%
UFL	Gainesville, Florida	327	3.14%
CHLA	Los Angeles, California	320	3.07%
UWM	Wauwatosa, Wisconsin	311	2.99%
SRI	Menlo Park, California	281	2.70%
ROC	Rochester, New York	207	1.99%

© 2023 Kim EH et al. *JAMA Network Open.*

eAppendix 2.6.2. Differences in demographics of cohort based on raw wearable device data availability

	Raw Wearable Device Data Availability			
	Overall	No	Yes	*P* value
**n**	7424	878	6546	
**Age (mean (SD))**	11.96 (0.72)	11.88 (0.74)	11.97 (0.71)	0.001
**Race/Ethnicity (%)**				<0.001
White	4301 (57.9)	251 (28.6)	4050 (61.9)	
Hispanic	1354 (18.2)	453 (51.6)	901 (13.8)	
Black	847 (11.4)	64 (7.3)	783 (12.0)	
Multiracial/ethnic	552 (7.4)	67 (7.6)	485 (7.4)	
AIAN/P	158 (2.1)	5 (0.6)	153 (2.3)	
Asian	137 (1.8)	26 (3.0)	111 (1.7)	
Other	75 (1.0)	12 (1.4)	63 (1.0)	
**Gender (%)**				0.906
Male	3839 (51.7)	449 (51.1)	3390 (51.8)	
Female	3571 (48.1)	428 (48.7)	3143 (48.0)	
Different/Gender Queer/Trans	9 (0.1)	1 (0.1)	8 (0.1)	
Don't know/Refused to answer	3 (0.0)	0 (0.0)	3 (0.0)	
**ADI percentile (mean (SD))**	38.30 (25.65)	28.57 (22.24)	39.61 (25.79)	<0.001
**BMI category (%)**				<0.001
Healthy	4514 (60.8)	473 (53.9)	4041 (61.7)	
Obesity	1253 (16.9)	193 (22.0)	1060 (16.2)	
Overweight	1129 (15.2)	160 (18.2)	969 (14.8)	
Missing	253 (3.4)	28 (3.2)	225 (3.4)	
Underweight	275 (3.7)	24 (2.7)	251 (3.8)	

© 2023 Kim EH et al. *JAMA Network Open.*

eReferences

1. Growth Charts - Homepage. Published January 31, 2019. Accessed June 13, 2022. https://www.cdc.gov/growthcharts/index.htm
2. UNESCO Institute for Statistics. *International Standard Classification of Education: ISCED 2011*. UNESCO Institute for Statistics; 2012. Accessed April 4, 2022. http://www.uis.unesco.org/Education/Documents/isced-2011-en.pdf
3. Fitabase - Research Device Data and Analytics. Accessed December 19, 2022. https://www.fitabase.com/
4. Kumar D, Klefsjö B. Proportional hazards model: a review. *Reliab Eng Syst Saf*. 1994;44(2):177-188. doi:10.1016/0951-8320(94)90010-8
5. Rich JT, Neely JG, Paniello RC, Voelker CCJ, Nussenbaum B, Wang EW. A practical guide to understanding Kaplan-Meier curves. *Otolaryngol Neck Surg*. 2010;143(3):331-336. doi:10.1016/j.otohns.2010.05.007

© 2023 Kim EH et al. *JAMA Network Open.*
