# Supplementary material for: Association of Demographic and Socioeconomic Indicators With the Use of Wearable Devices Among Children
Source: JAMA Netw Open. 2023 Mar 30;6(3):e235681. doi: 10.1001/jamanetworkopen.2023.5681 (PMC10064258; doi:10.1001/jamanetworkopen.2023.5681)
Supplement: Supplement 2. — Data Sharing Statement [file jamanetwopen-e235681-s002.pdf]

Data Sharing Statement

Kim. Association of Demographic and Socioeconomic Indicators With the Use of Wearable Devices Among Children. *JAMA Netw Open*. Published March 30, 2023.
doi:10.1001/jamanetworkopen.2023.5681

Data
Data available: Yes
Data types: Deidentified participant data, Data dictionary
How to access data: The source data and the associated data dictionaries from the ABCD Study are available to authorized data users at the ABCD Data Archive:
https://nda.nih.gov/data_dictionary.html?source=ABCD%2BRelease%2B4.0&submission=ALL
When available: beginning date: 10-27-2021

Supporting Documents
Document types: Statistical/analytic code
How to access documents: The complete code used for data loading and analysis is available through an open-source GitHub code repository -
https://github.com/aid4mh/ABCD_Fitbit_Retention_Analysis
When available: With publication

Additional Information
Who can access the data: https://nda.nih.gov/tutorials/abcd/query_and_download.html?chapter=querying-abcd
Types of analyses: per the terms and conditions of data usage agreement
Mechanisms of data availability: Signed data usage agreement
Any additional restrictions: NA
